# Structural Aspects of DNA Repair and Recombination in Crop Improvement

**DOI:** 10.3389/fgene.2020.574549

**Published:** 2020-09-11

**Authors:** Prabha Verma, Reetika Tandon, Gitanjali Yadav, Vineet Gaur

**Affiliations:** National Institute of Plant Genome Research, New Delhi, India

**Keywords:** DNA repair and recombination, photolyases, glycosylases, structure-specific endonucleases, transcriptomic interventions, crop improvement

## Abstract

The adverse effects of global climate change combined with an exponentially increasing human population have put substantial constraints on agriculture, accelerating efforts towards ensuring food security for a sustainable future. Conventional plant breeding and modern technologies have led to the creation of plants with better traits and higher productivity. Most crop improvement approaches (conventional breeding, genome modification, and gene editing) primarily rely on DNA repair and recombination (DRR). Studying plant DRR can provide insights into designing new strategies or improvising the present techniques for crop improvement. Even though plants have evolved specialized DRR mechanisms compared to other eukaryotes, most of our insights about plant-DRRs remain rooted in studies conducted in animals. DRR mechanisms in plants include direct repair, nucleotide excision repair (NER), base excision repair (BER), mismatch repair (MMR), non-homologous end joining (NHEJ) and homologous recombination (HR). Although each DRR pathway acts on specific DNA damage, there is crosstalk between these. Considering the importance of DRR pathways as a tool in crop improvement, this review focuses on a general description of each DRR pathway, emphasizing on the structural aspects of key DRR proteins. The review highlights the gaps in our understanding and the importance of studying plant DRR in the context of crop improvement.

## Introduction

Agriculture played an essential role in directing human evolution from hunter-gatherer to agro-pastoralist lifestyle (Hervella et al., 2012), which in turn resulted in changed feeding habits (Luca et al., 2010) and steep increase in population growth rates (Zahid et al., 2016). However, agriculture is now threatening various ecosystems (Foley et al., 2011; Tilman et al., 2011). The combined effect of exponentially increasing global population and concomitant increase in malnutrition has put considerable strains on agriculture. The green revolution in the 1960s considerably enhanced crop production (Conway and Toenniessen, 1999) but was limited to a few species and geographical regions. However, present-day crops are more vulnerable to stress, with greater dependence on chemical pesticides, and productivity is still unable to meet the demand. Therefore, agriculture needs a second revolution (Wollenweber et al., 2005) to increase productivity without increasing the cultivable land (Foley et al., 2011). Conventional breeding methods (Breseghello and Coelho, 2013) or an understanding of genetic engineering (Genetic modification and Genome editing) (Zhang et al., 2018) can assist in realizing these goals. Even though conventional plant breeding (hybridization and selection to achieve rearranging of the genome) is the preferred approach, but it is time-consuming and labor-intensive (Blary and Jenczewski, 2019; Zhang et al., 2018). On the other hand, technological advancement, and availability of gene sequences have enabled researchers to either insert a DNA sequence (genetic modification, GM) or precisely edit any gene sequence of the plant (genome editing). Coupling genetic modification and genome editing with conventional plant breeding can expedite the research for crop improvement.

Genetic modification involves the transfer of a foreign nucleic acid (transgenic, cisgenic, or intragenic) into a plant of economic importance resulting in generating an entirely new trait (*e.g.*, tolerance against various biotic and abiotic stresses) (Bawa and Anilakumar, 2013; Kamle et al., 2017). However, GM crops are associated with controversies of social, environmental, and human health-related aspects (Bawa and Anilakumar, 2013; Jones, 2015; Ali et al., 2019). Genome editing, in contrast, has emerged in recent years as a more acceptable alternative to transgenic modification since the introduced changes mimic natural changes to a large extent. Genome editing employs site-directed nucleases (Zinc finger nucleases (ZFNs), transcription activator-like effector nucleases (TALENs), and clustered regularly interspaced short palindromic repeats (CRISPR)/Cas system) to precisely make alterations in a pre-determined site in a sequence-specific manner to alter the function of the target gene (Voytas, 2013; Chen and Gao, 2014; Zhang et al., 2018). The basic principle of crop improvement using site-specific endonucleases relies on the generation of double-stranded breaks (DSBs), which are then repaired by the internal DNA repair and recombination (DRR) machinery of the plant itself (Symington and Gautier, 2011).

DNA can undergo damage due to various exogenous (Ionizing radiations, UV-radiations, alkylating agents) or endogenous (Intracellular metabolism and DNA metabolism) factors resulting in a variety of different DNA lesions as listed in Table 1. These DNA lesions, if not repaired, can result in impaired cellular processes, and lead to genome toxicity. Therefore, all organisms have evolved a range of DRR mechanisms. Plants being sedentary have further evolved DRR mechanisms (Singh et al., 2010) and, thus, an in-depth and objective study on DRR in plants is crucial. Among plants, DRR mechanisms operate in tissue-specific, developmentally regulated, and cell-cycle dependent manner. Some DRR mechanisms are antagonistic, while others are redundant with entirely different outcomes. Some DRR pathways are efficient, while others are inherently more error-prone. Plant DRR mechanisms constitute a delicately regulated process; they can slow down with the aging of plants (Britt, 1999; Hefner et al., 2006; Uchiyama et al., 2004; Manova and Gruszka, 2015). Many of these pathways play an essential role in DNA repair in somatic cells, whereas the same pathways play an important role in genetic recombination (Schuermann et al., 2005). Most of our understanding regarding DRR mechanisms in plants comes from structural and biochemical studies in prokaryotes, yeast, and animals. Plant DRR related genes and proteins have been identified through homology-based searches, and there is still a wide gap in their structural and biochemical studies. Therefore, the information about plant DRR is available only in bits and pieces.

**TABLE 1:** Types of DNA lesions and their repair mechanisms.

DNA lesion	Source	Repair mechanisms^#^
Mismatch lesion 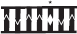	*Endogenous sources:*	MMR
	Nucleotide misincorporation during DNA Replication,	BER
		Homologous recombination,	
		Spontaneous hydrolytic deamination of DNA bases	
		*Exogenous sources:*	
		Oxidizing agents, Alkylating agents	
Single strand breaks 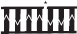	*Endogenous sources:*	BER
	Endogenous reactive oxygen species,	
		Enzymatic cleavage of phosphodiester bond during BER,	
		Abortive DNA Topoisomerase I activity	
		*Exogenous Sources:*	
		Ionizing radiations, Anti-cancer drugs	
Double strand breaks 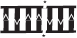	*Endogenous sources:*	HR
	Endogenous reactive oxygen species,	NHEJ
		DNA replication and repair, Meiosis I,	
		T-cell receptor formation	
		Immunoglobulin class switching	
		Excision of transposable element	
		*Exogenous Sources:*	
		Ionizing radiations, Radiomimetic compounds	
		Engineered nucleases	
DNA intrastrand crosslinks 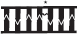	*Endogenous sources:*	Photoreactivation
	Reactive aldehydes,	NER
		Endogenous reactive oxygen species	TLS
		*Exogenous Sources:*	
		Ultraviolet radiation, Ionizing radiations,	
		Cisplatin, Mitomycin C	
DNA interstrand crosslinks 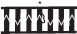	*Endogenous sources:*	NER
	Nitrous acid, Reactive aldehydes,	HR
		*Exogenous Sources:*	NHEJ
		Alkylating agents, Cisplatin,	TLS
		Mitomycin C, Psoralens	
DNA-Protein crosslinks 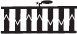	*Endogenous sources:*	NER
	Reactive aldehydes, metals,	HR
		Enzymatic DNA-Protein crosslinks	Crosslink
		*Exogenous sources:*	hydrolysis
		Ultraviolet radiation, Ionizing radiations,	Proteolysis
		Chemotherapeutic agents	
Base modification 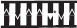	*Endogenous sources:*	BER
	Nitric oxide, Superoxide,	NER
		Spontaneous hydrolysis of N-glycosidic bond	TLS
		Spontaneous hydrolytic deamination of DNA bases	
		DNA methylation	
		*Exogenous sources:*	
		Ultraviolet radiation, Ionizing radiations,	
		Alkylating agents	

*^#^MMR, Mismatch Repair; BER, Base Excision Repair; HR, Homologous Recombination; NHEJ, Non-Homologous End Joining; NER, Nucleotide Excision Repair; TLS, Translesion DNA Synthesis. ^∗^Site of DNA damage.*

DRR is an important life process involved in the maintenance of genome stability and is equally vital for application-based work such as crop improvement. Regardless of the approach: traditional plant breeding or targeted, the success rate is mostly dependent upon the complex interplay between various DRR pathways (Manova and Gruszka, 2015). This review aims to provide an overview of different DRR pathways, with emphasis on scope and extent of available knowledge in the plant kingdom, as well as structural and biochemical aspects of various DRR mechanisms and their potential for crop improvement.

## DNA Repair and Recombination in Plants

**FIGURE 1:**
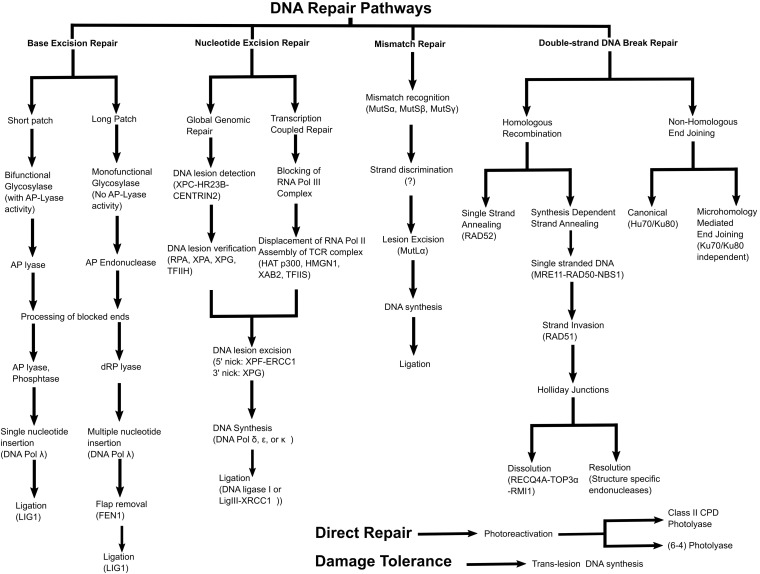
Summary of various DNA repair and recombination pathways.

Plants have achieved substantial specializations in their DNA repair and recombination methods compared to other living organisms due to their sedentary lifestyle and inability to avoid environmental factors that could ultimately result in DNA damage (Table 1). The main mechanisms of DRR in plants are direct repair (Photoreactivation), base excision repair (BER), nucleotide excision repair (NER), mismatch repair (MMR), non-homologous end joining (NHEJ), homologous recombination (HR), and translesion DNA synthesis (TLS) as shown in Figure 1 (Britt, 1999; Singh et al., 2010; Manova and Gruszka, 2015). Understanding of DRR mechanisms in plants comes from studies in other organisms (bacteria, yeast, and animals). The following section provides a brief account of the various mechanisms of DRR in plants and the differences exhibited by plant DRR compared to other living organisms.

### Direct Repair

Direct repair involves a direct reversal of DNA lesions by enzymatic reactions; therefore, it is an error-free pathway. Ultraviolet radiations are the most common DNA damaging agent. UV radiation mostly generates cyclobutane pyrimidine dimers (CPDs), and [6-4]pyrimidinone dimers (also called (6-4) photoproducts) (Britt, 1999). These lesions can be repaired either by light-dependent direct repair (photoreactivation) or by light-independent excision of the lesion (dark repair). Photoreactivation is carried out by a class of enzymes called photolyases, which shows activity in the presence of light (360–420 nm) (Brettel and Byrdin, 2010). Plants have two types of photolyases: Class II CPD photolyase and (6-4) photolyase. Photoreactivation is one of the well-studied mechanisms of DNA repair in plants (Maul et al., 2008; Hitomi et al., 2009, 2012). Spontaneously occurring photolyase variants are associated with differing plant growth and productivity (Hidema et al., 1997; Hitomi et al., 2012). AGT (O^6^-Alkylguanine-DNA-alkyltransferase) is another enzyme involved in the direct repair. AGT transfers the alkyl group of alkylated bases to a Cys residue of the enzyme in an irreversible reaction. Interestingly, till now, AGT homologs have not been found in plants (Pegg, 2011).

### Base Excision Repair (BER)

Base Excision Repair repairs DNA lesions resulting from oxidation, alkylation, or deamination of the nitrogenous base of DNA (Roldan-Arjona et al., 2019). The first step involves damage recognition and cleavage of N-glycosidic bond resulting in the generation of an abasic site (AP site) by DNA glycosylases. Spontaneous hydrolysis of the N-glycosidic bond can also generate an AP site (Sheppard et al., 2000). The AP site thus generated is further processed either by AP lyase (short patch BER) or AP endonuclease (long patch BER). Both AP lyase and AP endonuclease generate blocked ends that need to be processed further to facilitate the DNA polymerase and DNA ligase activities. Short patch BER inserts only one nucleotide, whereas long patch BER inserts multiple nucleotides resulting in a 5′ flap structure, requiring further processing by flap endonuclease (FEN1) before ligation. The mechanism of BER in plants is almost identical to other organisms with specific differences (Manova and Gruszka, 2015; Roldan-Arjona et al., 2019). Among mammals, DNA polymerase β and DNA ligase III play a significant role in short patch BER (Kubota et al., 1996). However, both these proteins are absent in plants. DNA polymerase λ may be participating in short patch BER in plants. The role of replicative polymerases in long patch BER among plants needs further scrutiny (Roldan-Arjona et al., 2019). In the absence of DNA ligase III (LIG 3) in plants, an ortholog of mammalian ligase I (*At*LIG1) play a role in both short patch as well as long patch BER (Cordoba-Canero et al., 2011).

### Nucleotide Excision Repair (NER)

Nucleotide Excision Repair is the most conserved DNA repair mechanism among all the eukaryotes. It primarily repairs UV induced DNA lesions and bulky DNA adducts. Based on the mode of DNA lesion identification, NER can operate as (1) Global genomic repair (repair influenced by a lesion-induced change in DNA structure) or (2) Transcription coupled repair (repair initiated by lesion-induced transcription inhibition). The NER in plants is like other eukaryotes (Xu et al., 1998; Gallego et al., 2000; Liu et al., 2000; Molinier et al., 2004; Canturk et al., 2016). The NER involves lesion recognition, verification, excision, DNA synthesis, and finally, ligation of the repaired stretch. In global genome repair, a heterotrimeric complex (XPC/HR23B/Centrin2, Table 2) detects DNA lesion as a local distortion in the DNA structure. Verification of the lesion and identification of the DNA strand harboring the lesion is carried out by a multi-protein complex (RPA/XPA/XPG/TFIIH, Table 2). Verification is followed by nicks on 5′ and 3′ sides of the lesion by XPF (Xeroderma pigmentosum, complementation group F)-ERCC1 (Excision Repair Cross-Complementation Group 1) and XPG (Xeroderma pigmentosum, complementation group G) nucleases, respectively. The downstream events involve DNA synthesis (DNA polymerases δ, ε, or κ) and ligation (DNA ligase I or DNA ligase III/XRCC1). Transcription coupled repair initiates when a DNA lesion in the template strand halts the progression of RNA Pol II complex. Repair machinery (HAT p300/HMGN1/XAB2/TFIIS, Table 2) displaces halted RNA Pol II complex. XPF-ERCC1 and XPG incise the lesion, followed by DNA synthesis and ligation. In addition to photoreactivation, players of NER are also important targets for enhancing UV tolerance among plants. There is a crosstalk between NER and HR through Centrin2. A defect in Centrin2, a key player in detecting DNA lesion in global genome repair, results in an enhanced somatic HR (Dubest et al., 2004; Molinier et al., 2004, 2008). Therefore, NER is one of the attractive targets for crop modification since it can be used to fine-tune the recombination frequency.

**TABLE 2:** Frequently used abbreviations in the article.

BER	Base Excision Repair
CRISPR	Clustered Regularly Interspaced Short Palindromic Repeats
DDM1	Decrease in DNA methylase 1
DRR	DNA Repair and Recombination
DSBs	Double Stranded DNA Breaks
EME1	Essential Meiotic Structure-Specific Endonuclease 1
ERCC1	Excision Repair Cross-Complementation Group 1
FEN1	Flap Endonuclease 1
GEN1	Flap Endonuclease GEN Homolog 1
HAT P300	Histone Acetyltransferase P300
HJs	Holliday Junctions
HMGN1	High Mobility Group Nucleosome Binding Domain 1
HR	Homologous Recombination
HR23B	Human homologue of Rad23B
iHJ	Intact Holliday Junction
Ku70	Lupus Ku autoantigen protein p70
Ku80	Lupus Ku autoantigen protein p80
LIG3	DNA Ligase 3
MET1	Methyltransferase 1
MMEJ	Microhomology Mediated End Joining
MMR	Mismatch Repair
MRE11	Meiotic Recombination 11
MUS81	MMS and UV Sensitive
nHJ	Nicked Holliday Junction
NBS1	Nibrin
NER	Nucleotide Excision Repair
NHEJ	Non-Homologous End Joining
PARP	Poly(ADP-Ribose) Polymerase
RECQ4A	RecQ helicase
RMI1	RecQ Mediated Genome Instability 1
RTR	RECQ4A-TOP3α-RMI1
SDNs	Site directed nucleases
SDSA	Synthesis-Dependent Strand Annealing
SEND1	Single-strand DNA endonuclease 1
SLX1	Synthetic Lethal of Unknown (X) function
SLX4	Synthetic Lethal of Unknown (X) function
SSA	Single-Strand Annealing
SSEs	Structure-Specific Endonucleases
TALENs	Transcription Activator-Like Effector Nucleases
TFIIH	Transcription factor II Human
TLS	Translesion DNA Synthesis
TOP3α	DNA Topoisomerase III Alpha
XAB2	XPA Binding Protein 2
XPA	Xeroderma pigmentosum, complementation group A
XPC	Xeroderma pigmentosum, complementation group C
XPF	Xeroderma pigmentosum, complementation group F
XPG	Xeroderma pigmentosum, complementation group G
XRCC1	X-Ray Repair Cross Complementing 1
ZFNs	Zinc Finger Nucleases

### Mismatch Repair (MMR)

Mismatch Repair corrects mismatches due to misincorporation of nucleotides during DNA replication and recombination. The prokaryotic MMR provides the basic MMR template for deciphering the molecular events involved in eukaryotic MMR (Harfe and Jinks-Robertson, 2000; Li, 2008). The essential players in prokaryotic MMR are homodimeric MutS (with an ATPase activity) (Lamers et al., 2003), homodimeric MutL (with an ATPase activity) (Spampinato and Modrich, 2000), and monomeric MutH (Ban and Yang, 1998). MutS identifies the lesion (mismatches and loops arising from insertion and deletion) and recruits MutL. MutL, in turn, recruits and activate MutH. MutH distinguishes the daughter strand from the parental strand by binding the nearest (either on 3′ or 5′ side) hemimethylated dGATC sequence to the lesion (Lahue et al., 1989). Binding of MutH is followed by an incision in the daughter strand and recruitment of UvrD (helicase II). UvrD unwinds the DNA duplex towards the mismatch lesion (Matson and Robertson, 2006). SSB (single-stranded DNA binding protein) protects the single-stranded DNA. Based on the location of incision relative to the lesion either a 3′-5′ exonuclease (ExoI or ExoX) or a 5′-3′ exonuclease (ExoVII or RecJ) removes the nicked strand. DNA Polymerase III and DNA ligase finally complete the repair process (Harfe and Jinks-Robertson, 2000; Li, 2008). Among eukaryotes, MutS has given rise to 6 genes (7 genes in plants) called MSH (MutS homologs) (Fishel and Wilson, 1997; Pochart et al., 1997; Culligan and Hays, 2000) and MutL gene gave rise to MLH (MutL homologs) and PMS (Post Meiotic Segregation) (Kolodner, 1996; Wang et al., 1999). In eukaryotes, mismatch recognition is carried out by MutSα (MSH2/MSH6 heterodimer), and MutSβ (MSH2/MSH3 heterodimer). Plants also have a third heterodimeric complex MutSγ (MSH2/MSH7). All these complexes have various overlapping substrate specificities (Wu et al., 2003; Emmanuel et al., 2006; Gomez and Spampinato, 2013; Chirinos-Arias and Spampinato, 2020). MutLα (MLH1/PMS2) heterodimer carries out the function of MutL. Unlike prokaryotic MutL, eukaryotic MutLα also has an endonuclease activity (Kadyrova and Kadyrov, 2016). Since eukaryotes lack methylation and MutJ homologs, strand-specific cleavage in eukaryotic MMR is not clear (Li, 2008). DNA re-synthesis and DNA ligation follow the excision of the lesion. It is noteworthy that, besides repair of mismatches, MMR repairs UV induced lesions and inhibits HR between divergent sequences, therefore, maintain the interspecies barrier (Tham et al., 2016). Correction of UV lesions and inhibition of HR have implications in the efficiency of methods used in crop improvement.

### Double-Stranded DNA Break Repair (DSB Repair)

Double-stranded DNA breaks (DSBs) are the most lethal type of DNA damages, and they are also the most important lesions in terms of crop-improvement. Two independent mechanisms repair DSBs: homologous recombination (HR) and non-homologous end joining (NHEJ). Repair of DSBs via HR depends upon the information from homologous sequences, whereas NHEJ does not require any sequence information and therefore is more error-prone compared to HR. In plants, NHEJ is more frequent than HR: a safeguard mechanism to prevent recombination between two non-allelic sequences (Puchta and Fauser, 2014; Manova and Gruszka, 2015).

Homologous recombination plays an essential role in DNA repair in somatic cells and the generation of diversity in meiotic cells (Schuermann et al., 2005). HR is also involved in repairing interstrand crosslinks and helps in the restart of the stalled replication fork (Li and Heyer, 2008; Manova and Gruszka, 2015). HR can proceed through one of the two mechanisms: (1) Single-strand annealing (SSA), and (2) synthesis-dependent strand annealing (SDSA) (Kimura and Sakaguchi, 2006; Li and Heyer, 2008; Manova and Gruszka, 2015; Wright et al., 2018). Repair by SSA takes place when DSB occurs in between homologous sequences with the help of RAD52. SSA involves the removal of non-homologous overhangs, and therefore, this process is error-prone but plays a significant role in molecular evolution (Puchta, 2005). SDSA, on the other hand, relies on homologous sequences present on the sister chromatid or homologous chromosome. SDSA initiates with the formation of a single-stranded DNA (MRN complex: MRE11/RAD50/NBS1, Table 2) (Akutsu et al., 2007) followed by strand invasion with the help of RAD51, resulting in the formation of a D-loop (Abe et al., 2005; Osakabe et al., 2005). One of the intermediates in this process is the formation of a four-way DNA junction, known as Holliday Junction (HJ). These HJs need to be processed into two independent DNA duplexes. The processing of HJs is carried out by two independent mechanisms: (1) dissolution: requiring action of helicase (RECQ4A), topoisomerase (TOP3α) and a structural protein RMI1 (RecQ Mediated Genome Instability 1) (Bagherieh-Najjar et al., 2005; Rohrig et al., 2016), and (2) resolution: requiring action of structure-specific endonucleases (SSEs) (Matos and West, 2014). Dissolution gives rise to non-crossovers, but resolution can give rise to both non-crossovers as well as crossovers depending upon how the SSEs make nick at the crossover point of HJs. SSEs have been characterized extensively in bacteria, yeast, and animals. The function of SSEs is not restricted to HJ resolution only; a few also participate in other DRR mechanisms like interstrand crosslink repair and NER. Sequence homology allowed the identification of various SSEs in plants (GEN1, SEND1, MUS81-EME1, SLX1, FEN1, XPF-ERCC1, Table 2). Many of the SSEs from plants need to be characterized further for an in-depth understanding of HR in plants (Table 3).

**TABLE 3:** A summary of key proteins and their functions that have potential to be used as tool kits for crop improvement.

Name of the protein	Name of the gene^#^	Protein family	Mutation/overexpression studies	Miscellaneous information	Structural information from plants*	References
**Photolyases (Potential use in increasing UV resistance)**						
Class II CPD photolyase	*OsPHR* (OSNPB_100167600) *PHR1* (AT1G12370)	PHR2 superfamily	*PHR1* mutants show no differences from wild type in the absence of UV light. In the presence of UV light, PHR*1* mutants show growth inhibition and leaf necrosis. *PHR1* overexpression resulted in enhanced DNA repair in *A. thaliana.*	Repair UV induced cyclobutane pyrimidine dimers. Essential for the survival in the presence of UV-B. Widespread expression in somatic tissues and during later flower development. No expression of *PHR1* in dark grown etiolated seedlings. More UV tolerant rice cultivars have Gln296 in class II CPD photolyase, while less tolerant cultivars have His at this position. Norin 1 is UV sensitive because of Gln to Arg change at position 126.	Class II CPD photolyase from *O. sativa* (PDB: 3UMV)	(Hidema et al., 2000) (Yamamoto et al., 2007) (Hitomi et al., 2012) (Willing et al., 2016) (Ahmad et al., 1997) (Landry et al., 1997) (Kaiser et al., 2009)
(6-4) photolyase	*OsUVR3* (OSNPB_020204400) *AtUVR3* (AT3G15620)	PhrB superfamily	A nonsense mutation results in photoreactivation defect	Repair UV induced (6-4) photoproducts. Expression of *AtUVR3* is downregulated by light in photosynthesis dependent manner. Structural studies show that local differences in the amino acids contribute toward the major functional differences presented by PHR/CRY family instead of large structural changes.	(6-4) photolyase from *A. thaliana* (PDB: 3FY4)	(Katarzyna Banas et al., 2018) (Hitomi et al., 2009) (Nakajima et al., 1998) (Jiang et al., 1997)
**DNA glycosylases (Potential use in increasing resistance to abiotic and biotic stresses and altering efficacy of targeted mutagenesis)**						
Uracil DNA glycosylase (UDG)	*AtUNG* (AT3G18630)	UDG superfamily	*AtUNG* mutant plants display a normal phenotype and increased resistance against 5-fluorouracil (5-FU) cytotoxicity.	Removes Uracil from DNA. Strict substrate specificity in contrast to bacterial or human enzymes.	No structure known. 54.55% identity with human UDG (PDB: 3TKB)	(Cordoba-Canero et al., 2010)
Alkylpurine DNA glycosylase (AAG)	*AtMAG* (AT3G12040)	AAG Superfamily	-	Removes alkylated purines. A role in DNA replication and cell growth. High expression of *AtMAG* in rapidly dividing tissues and growing leaves.	No structure known.	(Santerre and Britt, 1994) (Shi et al., 1997)
8-oxoguanine-DNA glycosylase 1 (OGG1)	*AtOGG1* (AT1G21710)	HhH Superfamily	*AtOGG1* overexpression improves seed longevity and enhances abiotic stress tolerance. *AtOGG1* mutants show no phenotypic differences in comparison to the wild type.	Removes 7,8-dihydro-8-oxoguanine (8-oxo-G) from DNA.	No structure known. 38.91% identity with human 8-oxoguanine-DNA glycosylase (PDB: 1KO9)	(Chen et al., 2012) (Morales-Ruiz et al., 2003) (Garcia-Ortiz et al., 2001) (Dany and Tissier, 2001) (Murphy, 2005)
Methyl-CPG-binding domain 4-like (MBD4L)	*AtMBD4* (AT3G63030)	HhH Superfamily	*AtMBD4* overexpression induces the expression of LIG1 (also involved in BER). *AtMBD4* overexpression enhances tolerance to oxidative stress. Mutation at *AtMBD4* locus exhibits altered root architecture.	Removes uracil and thymine mispaired with G. CpG sequence context preferred. AtMBD4L lacks the signature MBD domain but possesses a conserved glycosylase domain important for conferring substrate specificity. *AtMBD4* expresses in perivascular leaf tissues, flowers, and the apex of immature siliques. AtMBD4L negatively regulates a subset of phosphate starvation genes.	No structure known. 34.43% identity with human MBD4 (PDB: 3IHO)	(Ramiro-Merina et al., 2013) (Nota et al., 2015) (Parida et al., 2019)
Endonuclease III (NTH)	*AtNTH1* (AT2G31450) *AtNTH2* (AT1G05900)	HhH Superfamily	-	AtNTH1 and AtNTH2 are structural and functional homologues of *E. coli* endonuclease III and protects plant against oxidative damages. AtNTH1 and AtNTH2 appear to be targeted to the chloroplast.	No structure known. 32.24% (AtNTH1) and 30.85% (AtNTH2) identity with *E. coli* endonuclease III (PDB: 2ABK)	(Gutman and Niyogi, 2009) (Roldan-Arjona et al., 2000)
Demeter (DME)	*AtDME* (AT5G04560)	DML family of HhH Superfamily	Loss-of-function mutations in *AtDME* cause seed abortion. Knocking down *AtDME* expression in a triple mutant background (*ros1 dml2 dml3*) has enhanced disease susceptibility to *Fusarium oxysporum* infection.	Excises 5-methylcytosine (5-meC) from DNA. DME shows a preference for 5-meC over thymine in CpG sequence context. Regulates genomic imprinting through its interaction with histone H1. Active DNA demethylation by DME also requires HMG domain containing SSRP1. DME is required for endosperm gene imprinting and seed viability. DME demethylates similar kind of genes in both vegetative and central cells in male and female gametophytes.	No structure known. 30.86% identity with *Deinococcus radiodurans* endonuclease III (PDB: 1ORN)	(Rea et al., 2012) (Morales-Ruiz et al., 2006) (Schumann et al., 2019) (Ikeda et al., 2011) (Choi et al., 2002) (Gehring et al., 2006) (Ohr et al., 2007) (Schoft et al., 2011)
Repressor of silencing 1 (ROS1)	*AtROS1* (AT2G36490)	DML family of HhH Superfamily	*AtROS1* overexpression increases demethylation of promoter and coding regions of the genes involved in flavonoid biosynthesis and antioxidant pathways under salt stress. In *AtROS1* mutant plants many of the CpXpG and CpXpX sites become heavily methylated. ROS1 mutation causes transcriptional silencing of many specific genes.	ROS1 shows a preference for 5-meC over thymine in CpG sequence context. ROS1 preferentially targets transposable elements and intergenic regions. ROS1 positively regulates stress responsive genes. ROS1 regulates seed dormancy by negatively regulating DOGL4. ROS1 has been implicated in immune responsiveness of Arabidopsis. ROS1 antagonizes RNA dependent DNA methylation. Expression of ROS1 is promoted by DNA methylation and antagonizes by DNA demethylation. ROS1 is a slow-turnover enzyme, a feature that helps avoiding generation of double stranded breaks. ROS1 interacts with RPA2 during DNA repair. DNA damage binding protein (DDB2) is a critical regulator of ROS1 activity.	No structure known. 25.27% identity with *Deinococcus radiodurans* endonuclease III (PDB: 1ORN)	(Morales-Ruiz et al., 2006) (Tang et al., 2016) (Zhu et al., 2018) (Bharti et al., 2015) (Williams et al., 2015) (Ponferrada-Marin et al., 2009) (Zhu et al., 2007) (Agius et al., 2006) (Kapoor et al., 2005b) (Gong et al., 2002) (Zhu et al., 2018) (Lopez Sanchez et al., 2016) (Yamamuro et al., 2014) (Li et al., 2012) (Xia et al., 2006) (Kapoor et al., 2005a) (Cordoba-Canero et al., 2017) (Le et al., 2014)
DME like (DML)	*DML2* (AT3G10010) *DML3* (AT4G34060)	DML family of HhH Superfamily	Mutations in *DML2* and/or *DML3* lead to hypermethylation of cytosines that are normally unmethylated or weakly methylated, and hypomethylation of cytosines that are generally hypermethylated.	DML2 and DML3 are 5-meC DNA glycosylases. DML2 and DML3 play a role in maintaining methylation marks. DML2 and DML3 along with ROS1 play a role in resistance against fungal diseases.	No structure known.	(Ortega-Galisteo et al., 2008) (Le et al., 2014) (Brooks et al., 2014)
Formamido- pyrimidine DNA glycosylase (FPG)	*AtFPG1* (AT1G52500)	H2TH superfamily	Mutation in *AtFPG1* is compensated by *AtOGG1* and *vice versa*. A double mutant result in an increase in oxidative damage.	AtFPG1 excises oxidatively modified purines. Presence of a protein segment (213-229) enables AtFPG1 to process 8-oxoG in addition to other oxidative modifications. *AtFPG1* produces two transcripts arising from alternate splicing and encoding two proteins: AtFPG-1 and AtFPG-2. The two proteins exhibit differences in cleaving double stranded oligonucleotide containing 8-oxoG.	AtFPG1 (PDB: 3TWL, 3TWK)	(Duclos et al., 2012) (Gao and Murphy, 2001) (Murphy and Gao, 2001) (Cordoba-Canero et al., 2014)
**Structure Specific Endonucleases (Potential use in assisting site-directed nucleases)**						
FEN1	*SAV6* (AT5G26680) *OsFEN-1a* (OSNPB_050540100) *OsFEN-1b* (OSNPB_030834000)	Rad2/XPG	FEN1 mutation causes hypersensitivity to methyl methanesulfonate, UV-C and reduced telomere length. No effect of FEN1 mutation on chemicals that block DNA replication.	FEN1 removes 5′ flaps and important for maintaining genome stability. FEN1 is abundant in tissues rich in proliferating cells. FEN1 in plants either lacks or exhibits a weak exonuclease activity. Rice possess two FEN1 homologs: OsFEN1a and OsFEN1b. OsFEN1 physically interacts with OsPCNA.	No structure known. 54.69% identity with human FEN1 (PDB: 1UL1)	(Zhang J. et al., 2016) (Zhang Y. et al., 2016) (Kimura et al., 2003) (Kimura et al., 2001) (Kimura et al., 2000) (Kimura et al., 1997)
GEN1	*AtGEN1* (AT1G01880) *OsGEN-L* (OSNPB_090521900)	Rad2/XPG	Silencing of *OsGEN-L* results in low fertility, male-sterility, absence of mature pollens. Loss of function also results in persistent double strand breaks resulting in programmed cell death of the male gametes.	Like other eukaryotic GEN1, plant GEN1 is also a canonical Holliday junction resolvase. There are indications of sequence specificity exhibited by AtGEN1. *OsGEN-L* plays a role in early microspore development in rice.	No structure known. 31.35% identity with human GEN1 (PDB: 5T9J)	(Moritoh et al., 2005) (Wang et al., 2017) (Bauknecht and Kobbe, 2014)
SEND1	*AtSEND1* (AT3G48900) *OsSEND1* (OSNPB_080101600)	Rad2/XPG	Depletion of *OsSEND1* has no effect on plant development. *OsSEND1* depletion does not enhance the defect caused by *OsGEN1* depletion. Combined absence of *AtMUS81* and *AtSEND1* results in developmental defects, spontaneous cell death, and genome instability.	Like other eukaryotic GEN1, plant SEND1 is also a canonical Holliday junction resolvase. There are indications of sequence specificity exhibited by AtSEND1. AtSEND1 is as an essential backup to MUS81.	No structure known. 28.71% identity with human GEN1 (PDB: 5T9J)	(Wang et al., 2017) (Bauknecht and Kobbe, 2014) (Olivier et al., 2016) (Furukawa et al., 2003)
MUS81	*AtMUS81* (AT4G30870) *OsMUS81* (OSNPB_010948100)	XPF	Mutation in *AtMUS81* shows normal growth and no meiotic impairment because of the presence of alternative parallel pathways. Disruption of *AtMUS81* increases sensitivity to DNA damaging agents and moderate decrease in meiotic recombination. Mutation of *AtMUS81* in combination with mutation in *AtRecQ4A* results in synthetic lethality. This synthetic lethality can be suppressed by disrupting *RAD51C*.	MUS81 is the catalytic partner in MUS81-EME1 complex. MUS81-EME1 plays an important role in meiotic DNA damage and meiotic recombination in addition to somatic DNA repair and recombination. It is also an important player in interstrand cross-link repair. *AtMUS81* transcript is present in all tissues, but almost 9-fold higher concentration in anthers. *OsMUS81* gene produces two alternative transcripts.	No structure known. 32.12% identity with human MUS81 (PDB: 4P0P)	(Kurzbauer et al., 2018) (Higgins et al., 2008) (Berchowitz et al., 2007) (Hartung et al., 2006) (Mannuss et al., 2010) (Mimida et al., 2007)
EME1	*AtEME1A* (AT2G21800) *AtEME1B* (AT2G22140) *OsEME1* (OSNPB_040648700)	XPF	-	EME1 is the non-catalytic partner in MUS81-EME1 complex. MUS8-EME1 plays an important role in meiotic DNA damage and meiotic recombination in addition to somatic DNA repair and recombination. It is also an important player in interstrand cross-link repair. Arabidopsis has two EME1 homologs: AtEME1A and AtEME1B. Both the homologs form distinct complexes with MUS81: MUS81-EME1A and MUS81-EME1B. Both the complexes have same cleavage patterns on DNA substrates with slight differences in processing intact Holliday junctions.	No structure known.	(Kurzbauer et al., 2018) (Higgins et al., 2008) (Berchowitz et al., 2007) (Hartung et al., 2006) (Mannuss et al., 2010) (Mimida et al., 2007) (Geuting et al., 2009)
SLX1	*T9D9.16* (AT2G30350)	GIY-YIG	-	Also known as HIGLE. Interacts with HYL1 and Serrate proteins.	No structure known. 22.61% identity with *Candida glabrata* SLX1 (PDB: 4XLG)	(Cho et al., 2017)
**Holliday Junction dissolution (potential use in manipulating the frequency of HR)**						
RecQ helicase	*AtRECQ4A* (AtRECQ4A) *AtRECQ4B* (AT1G60930) *OsRECQL4* (OSNPB_040433800)	Superfamily II DNA helicases	*OsRECQL4* and *AtRECQ4A* mutant plants are hypersensitive to DNA damaging agents and exhibit high HR frequency. *AtRECQ4B* mutant plants are not mutagen sensitive but can impair HR (antagonistic to *AtRECQ4A)*. A double mutant for *AtRecQ4A* and *AtMUS81* is lethal. Mutation of *AtRecQ4A* but not *AtRecQ4B* can suppress the lethal phenotype of *AtTop3α* mutant.	AtRecQ4A plays a role in removing inter-chromosomal telomeric connections during meiotic recombination.	No structure known. 40.83% (AtRECQ4A) and 38.64% (AtRECQ4B) identity with human ATP dependent helicase Q1 (PDB: 4U7D)	(Kwon et al., 2013) (Hartung et al., 2007a) (Hartung et al., 2006) (Higgins et al., 2011)
TOP3α	*AtTOP3alpha* (AT5G63920)	Type IA topoiso- merase	Disruption of *AtTOP3alpha* results in fragmented chromosomes and sensitivity to camptothecin. Mutation of *AtRecQ4A* can suppress the lethal phenotype of *AtTop3α* mutant.	Essential for meiotic recombination. A defect in TOP3α results in sterile flowers.	No structure known. 43.86% identity with human Topoisomerase3α (PDB: 4CGY)	(Hartung et al., 2008) (Seguela-Arnaud et al., 2015)
RMI1	*AtRMI1* (AT5G63540)	OB-fold proteins	Disruption of *AtRMI1* exhibit phenotype like the disruption of *AtTOP3alpha.*	RMI1 is a structural protein. RMI1 is essential of meiotic recombination in plants. A role in DNA cross-link repair has been implicated.	No structure known. 40.00% identity with human RMI1 (PDB: 4CGY)	(Hartung et al., 2008) (Bonnet et al., 2013) (Chelysheva et al., 2008)
**Translesion DNA synthesis (Potential use in increasing UV tolerance)**						
Polη	*AtPOLH* (AT5G44740)	Y- family DNA polymerase	*AtPOLH* overexpression increases UV resistance. Disruption of *AtPOLH* increases mutation frequency.	Alternative splicing was detected. Expresses ubiquitously in plants.	No structure known. 41.20% identity with human Polη (PDB: 4O3S).	(Nakagawa et al., 2011) (Jesus Santiago et al., 2008) (Santiago et al., 2009) (Santiago et al., 2008) (Santiago et al., 2006)
Polκ	*AtPOLK* (AT1G49980)	Y- family DNA polymerase	-	Deletion of 193 amino acids from C-terminal markedly increases the activity and processivity of Polκ. Highly expressed in variety of tissues.	No structure known. 52.94% identity with human Polκ (PDB: 2OH2)	(Garcia-Ortiz et al., 2007) (Garcia-Ortiz et al., 2004)
Rev3	*AtREV3* (AT1G67500)	B- family DNA polymerase	Disruption of *AtREV3* decreases mutation frequency Disruption of *AtREV3* causes hypersensitivity to UV-B and gamma rays.	Catalytic subunit of Polζ. Rev3 cooperates with MUS81 in response to interstrand crosslinks and alkylated bases.	No structure known. 28.30% identity with human Polδ (PDB: 6S1M)	(Nakagawa et al., 2011) (Kobbe et al., 2015) (Sakamoto et al., 2003)
Rev7	*AtREV7* (AT1G16590)	HORMA (Hop1, Rev7 and Mad2) family	*AtREV7* disrupted mutant is sensitive to DNA cross-linker but no sensitivity toward UV-B and gamma rays.	Non-catalytic subunit of Polζ	No structure known. 33.33% identity with human MAD2B (PDB: 6BI7)	(Takahashi et al., 2005)
Rev1	*AtREV1* (AT5G44750)	Y- family DNA polymerase	Disruption of *AtREV1* decreases mutation frequency. *AtREV1* disrupted mutant is moderately sensitive to UV-B, gamma rays, and DNA cross-linkers. Plants with moderate over-expression of *AtREV1* could be obtained indicating toxic nature of Rev1 at high levels.	Alternative splicing was detected. Expresses ubiquitously in plants. AtRev1 is a deoxycytidyl transferase.	No structure known. 31.23% identity with human REV1 (PDB: 3GQC)	(Santiago et al., 2009) (Nakagawa et al., 2011) (Santiago et al., 2008) (Takahashi et al., 2005) (Jesus Santiago et al., 2008) (Takahashi et al., 2007)
Polι	Not identified	−	−	−	−	−

^#^Values in parentheses are locus tags. Only genes from Arabidopsis thaliana (At) and Oryza sativa (Os) are included. *Closest protein for which structure is available. Percentage sequence identity refers to sequence identity between A. thaliana proteins as query sequence and the non-plant proteins for which crystal structures are available.

Non-homologous end joining repairs DSBs along with HR. Even though NHEJ is sequence-independent and error-prone, it is still the most efficient method of DSB repair among plants (Puchta, 2005). One of the applications of NHEJ is during the integration of T-DNA into the genome during transformation (Park et al., 2015). In the canonical method of NHEJ, Ku70/Ku80 heterodimers bind to DSB ends to prevent further degradation and bring them in proximity. In mammals, Ku70/Ku80 (Lupus Ku autoantigen protein p70/Lupus Ku autoantigen protein p80) recruits DNA-PKcs (DNA dependent protein kinases), followed by the action of nucleases, DNA polymerases, and ligases. *At*KU70, *At*KU80, LIG4 proteins have been found in plants, while DNA-PKc kinase has not been identified so far (Nishizawa-Yokoi et al., 2012). A competing Ku70/Ku80 independent, alternate NHEJ pathway, MMEJ (Microhomology mediated end joining), is also known to repair DSBs. MMEJ utilizes microhomologous sequences at the DNA ends (Wang and Xu, 2017). MMEJ requires removal of flap strands after microhomology based sequence alignment, followed by ligation. Since this method involves trimming of the ends of DNA, MMEJ is highly mutagenic. The mechanism involves PARPs (Poly(ADP-Ribose) Polymerases), XRCC1, XPF, MRE11, LIG3 (Jia et al., 2013).

### Translesion DNA Synthesis (TLS)

Photoreactivation, BER, NER, or MMR take care of lesions arising from UV exposure or oxidative damage. However, all these processes are not capable of completely removing these lesions. Any of these lesions left behind stalls the progression of replicative DNA polymerase. A stalled replication fork can cause genome toxicity. However, plants and other organisms have evolved mechanisms to restart these stalled replication forks by bypassing the DNA lesions. Bypassing of the DNA lesions involves the removal of replicative DNA polymerase and binding of specialized DNA polymerases called TLS polymerases (Lehmann et al., 2007). TLS polymerases are known to have a spacious active site to accommodate bulky DNA lesions (Plosky and Woodgate, 2004). TLS polymerases mostly belong to the Y-family of DNA polymerases. These polymerases have been extensively studied biochemically and structurally in bacteria, yeast, and humans. Most of the TLS polymerases from plants (DNA polymerase ζ, η, κ, and Rev1) need to be characterized structurally and biochemically (Kimura and Sakaguchi, 2006; Roldan-Arjona and Ariza, 2009) (Table 3).

## Crop Improvement: Present Techniques and Futuristic Approaches

Genetic diversity is necessary for crop improvement to generate novel combinations of genes to achieve desired phenotypes (Glaszmann et al., 2010). A crop genome accumulates spontaneous mutations (reactive oxygen species, replication errors, transposable elements, ionizing radiation, *etc.*), therefore contributing diversity in the already existing genetic pool. However, the process is too slow to keep pace with ever-increasing demand. Meanwhile, many present-day crop plants have lost genetic diversity, compelling the intervention of crop improvement methods (Esquinas-Alcazar, 2005). Different crop improvement approaches are in use. These methods broadly fall into three categories: (1) chemical and physical mutagenesis, (2) transgenics, and (3) genome editing (Table 4). In the past few decades, these approaches have been used successfully for improving various traits of economically important plants: resistance to biotic and abiotic stresses, seed quality, crop yield, *etc.* and resulted in many fruitful outcomes. Intriguingly, DRR takes the central stage in all the crop improvement techniques.

**TABLE 4:** Comparison of various crop improvement techniques.

	Chemical/Physical mutagen	Transgenics	Genome editing
Mechanism	Use of chemical mutagens or ionizing radiations to induce random mutations.	*Agrobacterium tumefaciens* mediated transformation or gene gun (microprojectile bombardment) to introduce a foreign gene.	Use of site-directed nucleases (SDNs) to introduce DSBs
Outcome	Random mutations, mostly recessive in nature.	Introduction of a foreign gene through random insertion. Mostly resulting in a dominant trait.	Site-specific mutations through insertions or deletions. The introduced changes could be loss of function or gain of function.
DRR pathways involved	Base excision repair Nucleotide excision repair Translesion DNA synthesis Mismatch repair Non-homologous end joining Homologous recombination	Double-strand break repair Single-strand gap repair	Non-homologous end joining (SDN1) Homologous recombination (SDN2 and SDN3)
Advantages	Easiest method of introducing random mutations. Excluded from GMO regulations.	Introduces novel traits. A tool to study gene functions (loss of function/gain of function)	Site-directed. Partially exempted from strict GMO regulations.
Limitations	Obtaining a mutation of interest is dictated by chance event. Large population size is required for inducing mutation. Robust screening methodologies are required.	Environment and health safety concerns. Strong GMO regulations.	Tailor-made site-specific nuclease required. Generation of off-site cleavages.
Screening Methods	DNA markers, TILLING, HRM	PCR based detection methods, phenotypic characterization, DNA sequencing, ELISA	PCR based methods, Sanger sequencing, ELISA, MALDI-TOF, DNA Microarray, NMR
Scope of improvement	Mutations in mismatch repair pathway or overexpression of certain translesion DNA polymerase can reduce the need of using chemical or physical mutagens to introduce random mutations.	The efficiency of gene targeting can be increased by manipulating the efficacy of Homologous recombination.	Since the outcome of this technique rely on the mechanisms of DSB repair, a better control over NHEJ and HJ could open enormous application possibilities.

### Chemical and Physical Mutagenesis

One of the earliest methods for generating genetic diversity is inducing random mutations through ionizing radiations (*e.g.*, X-ray, γ-rays, neutron, and high-energy ion beams) or chemical mutagens (*e.g.*, alkylating agents, dyes, nitrous acid, *etc*.) (Shu et al., 2012; Holme et al., 2019). These methods result in random double-strand breaks, single-strand breaks, or base modifications, ensuing repair through specialized DRR mechanisms (Tables 1, 4). DRR mechanism thus activated dictates the outcome of mutagenesis: substitution or deletion (Oladosu et al., 2016). The advantage of this method is the cost-effectiveness, no need for the prior knowledge of gene function or sequence, and the technique is beyond the purview of GMO regulations. This method generates random mutations. Therefore, obtaining a mutation of interest is governed by chance events, pausing a significant limitation in terms of larger population size for mutagenesis and a robust screening methodology. The generation of chimeras is another limitation of this methodology in the case of vegetatively propagated plants (Geier, 2012). *In vitro* mutagenesis (mutation induced by treating an explant) and high-throughput mutation screening techniques (DNA molecular markers, TILLING: Targeting Induced Local Lesion In Genomes, HRM: High Resolution Melting, EMAIL: Endonucleolytic Mutation Analysis by Internal Labeling, *etc.*) overcame these limitations (Parry et al., 2009; Chen et al., 2014; Oladosu et al., 2016; Simko, 2016). This method has resulted in crops with various improved traits, *e.g.*, enhanced nutritional traits of soybean (Espina et al., 2018), agronomic traits of rice (Wu J.L. et al., 2005), biotic resistance in wheat (Hussain et al., 2018), *Medicago truncatula* seed size improvement (Ge et al., 2016), *etc*.

### Transgenics (Plant Transformation)

Transgenics involves introducing a foreign gene of a known function into the genome of a plant cell followed by the selection of transformed cells and, eventually, regenerating an entire transgenic plant. Transgenics, therefore, results in a genetically modified plant (Newell, 2000). Many transgenic plants have been created, since the mid-1980s (Caplan et al., 1983), using two techniques: *Agrobacterium tumefaciens* mediated transformation (Hwang et al., 2017) or gene gun (microprojectile bombardment) mediated transformation (Klein et al., 1992). Unlike chemical or physical mutagenesis, transgenics result in dominant traits. This method’s main advantage is the flexibility of introducing a gene of known function into a host plant. The gene often gets integrated randomly with the host genome either through microhomologies-mediated double-strand break repair or single-strand gap repair (Tzfira et al., 2004). Despite unlimited applications, strict GMO regulations due to environmental (invasiveness, intraspecific, and interspecific hybridization) and health (food toxicity and allergenicity) biosafety concerns (Stewart et al., 2000), are the significant limitations. Developing a genetically modified crop is therefore expensive as every GM crop must be assessed for environmental and health biosafety (Giraldo et al., 2019). Transgenics resulted in crops with improved traits, *e.g.*, nutritional value (Broun et al., 1999; Hirschberg, 1999), tolerance to various abiotic stresses (Holmberg and Bulow, 1998; Koh et al., 2007), herbicide resistance (Anthony et al., 1999), insect resistance (Dempsey et al., 1998), modified flower color (Mol et al., 1999), *etc*. Transgenics also has immense pharmaceutical potential in generating human therapeutic proteins in plants (Rodgers et al., 1999; Staub et al., 2000).

### Genome Editing (Targeted Mutagenesis)

Genome editing overcomes the disadvantages of random mutagenesis by physical and chemical mutagens. Since the changes introduced by genome editing mimic the natural changes, it is more acceptable than transgenics. Therefore, genome editing is the most promising technology for crop improvement in the current scenario (Abdallah et al., 2015). Genome editing relies upon tailor-made site-directed nucleases (SDN): ZFNs, TALENs, and CRISPR/Cas system. These nucleases make precise nicks in a sequence-specific manner resulting in DSBs. The outcome of genome editing relies upon the mode of DSB repair, which can either proceed through NHEJ or HR. NHEJ can result in the insertion or deletion of nucleotides (SDN1), while HR can facilitate the exchange of DNA sequence with an exogenously provided donor DNA containing the desired sequence/mutation (SDN2 and SDN3). The primary limitations of genome editing are off-target cleavages, a prerequisite knowledge of genomic information, and an efficient delivery method (Podevin et al., 2013). Genome editing successfully generated herbicide tolerance in maize (Shukla et al., 2009) and tobacco (Townsend et al., 2009), improved quality of soybean oil (Haun et al., 2014), stress tolerance in maize (Shi et al., 2017), improved yield traits in tomato (Soyk et al., 2017), *etc*. While most genome editing proceeds through NHEJ resulting in gene knockouts, genome editing’s true potential in the future relies on HR to generate traits that are difficult to achieve through conventional breeding (Abdallah et al., 2015).

Even though DRR is central to all crop improvement techniques, more studies on plant DRR mechanisms are important to innovate the crop improvement methodologies. Studying various DRR proteins from at least five different classes can greatly contribute toward developing new approaches for crop improvement: photolyases, DNA glycosylases, RTR complex, structure-specific endonucleases, and TLS DNA polymerases (Table 3). The knowledge of DRR can contribute toward crop improvement in four possible ways: (1) targeting DRR genes directly, (2) manipulation of HR frequency, (3) modification of gene-editing techniques, and (4) Computational systems biology and precision agriculture.

### Targeting DRR Genes Directly

Many of the DRR genes are known to be associated with biotic and abiotic stresses, and merely targeting these genes by altering their expression or protein structure might help generating improved crop plants (Table 3). Photolyases and many TLS DNA polymerases are associated with UV tolerance. Structure-based studies highlighted the importance of single amino acid substitution in stabilizing the overall structure of photolyase and subsequently affecting the UV sensitivity of the plant (Landry et al., 1997; Hidema et al., 2000; Yamamoto et al., 2007; Hitomi et al., 2012). Overexpression of CPD photolyase in *A. thaliana* resulted in increased DNA repair and enhanced UV tolerance (Kaiser et al., 2009). Altering the expression of TLS polymerases in *A. thaliana* showed an influence on UV tolerance. An overexpression of *AtPOLH* (coding Polη) increases the UV resistance (Jesus Santiago et al., 2008; Nakagawa et al., 2011), disruption of *AtREV3* (coding the catalytic subunit of Polζ) causes hypersensitivity to UV-B and γ-rays (Sakamoto et al., 2003), and disruption in *AtREV1* (coding REV1) moderately increases the sensitivity to UV-B and γ-rays (Jesus Santiago et al., 2008; Nakagawa et al., 2011; Table 3). Structural and biochemical characterization of TLS polymerases, similar to photolyases, are essential to understand the structural basis of repair of photoproducts and UV tolerance among plants in order to target these proteins for crop improvement. However, overexpressing TLS polymerase has its limitations since these polymerases are highly error-prone, and an overexpression might result in an increase in mutation frequency (Jesus Santiago et al., 2008; Nakagawa et al., 2011). Many DNA glycosylases play an essential role in tolerating oxidative stress. Overexpression of *AtOGG1* (codes for AtOGG1 DNA glycosylase) improves seed longevity (Chen et al., 2012), and overexpression of *AtMBD4* (codes for MBD4L DNA glycosylases) enhances tolerance to oxidative stress (Nota et al., 2015). Overexpression of *AtROS1* (codes for ROS1) activates the expression of genes coding for antioxidant pathways under salt stress (Bharti et al., 2015). DNA glycosylases belonging to the DEMETER family are critical for resistance against fungal diseases (Le et al., 2014; Schumann et al., 2019). The studies carried out in Arabidopsis provide clues about altering the expression of DNA glycosylases to improve tolerance against biotic and abiotic stresses. Similar studies need to be carried out in the crop plants to utilize the full potential of DNA glycosylases in crop improvement against various biotic and abiotic stresses.

### Manipulation of Homologous Recombination Frequency

Homologous recombination is the fundamental driving force behind generating diversity and new allelic combinations. An in-depth understanding of HR and its crosstalk with other DRR mechanisms can provide a robust tool for controlling HR events as and when required during a crop improvement program. Besides, it will also provide tools to facilitate homeologous recombination between divergent sequences. Any HR event starts with the generation of DSBs. During meiosis generation of DSBs is programmed and mediated by SPO11 proteins (Grelon et al., 2001; Hartung et al., 2007b). HR and NHEJ are the two competing pathways involved in DSB repairs. In fact, in plants, NHEJ (more error-prone) is the preferred DSB repair mechanism (Puchta, 2005). Two competing processes further reduce the generation of crossover species: HJ dissolution by RTR complex and HJ resolution by structure-specific endonucleases (Knoll et al., 2014). MMR also suppresses the frequency of HR (Tam et al., 2011). Furthermore, HR is developmentally regulated in plants (Boyko et al., 2006). Therefore, tinkering with NHEJ, processing of HJs, and MMR can increase the overall frequency of HR. Suppression of Ku70/Ku80 or LIG4 resulted in enhanced HR (Nishizawa-Yokoi et al., 2012). *AtRECQ4A* and *OsRECQL4* knockouts result in high HR frequency in Arabidopsis and rice, respectively (Hartung et al., 2007a; Kwon et al., 2013). Targeting MMR in plants is another very promising avenue to increase the frequency of HR and to enable homeologous recombination among related species. The loss of key MMR proteins (MSH2, MSH7, and PMS1) in Arabidopsis and tomato, increased homeologous recombination between divergent sequences (Emmanuel et al., 2006; Li et al., 2006, 2009; Tam et al., 2011). Besides being antagonistic to HR, MMR is a crucial player in maintaining genomic integrity. Arabidopsis with defective MMR proteins show a significant increase in the number of single-nucleotide variants in the gene. Therefore, MMR mutant plants could be used to introduce random mutations, thus replacing the need for chemical and physical mutagens (Hoffman et al., 2004; Van Marcke and Angenon, 2013; Belfield et al., 2018). Not all regions of a chromosome undergo HR with equal frequency. HR hotspots are mostly concentrated in euchromatin compared to heterochromatin (also comprises of functional genes). Therefore, DNA demethylation could be an approach to promote HR in heterochromatin regions. MET1 (Methyltransferase 1) and DDM1 (Decrease in DNA methylation 1) are involved in CG methylation maintenance. A loss of MET1 or DDM1 can restructure the distribution of crossing over hot spots along the chromosomes (Melamed-Bessudo and Levy, 2012; Yelina et al., 2012, 2015; Choi, 2017). The main limitation of any of these approaches will be to restore the silenced pathways since they are essential in maintaining genome stability.

### Modification of Genome Editing Techniques

Site-directed nucleases (SDN) revolutionized the field of genome editing. There are three different site-directed nuclease techniques: SDN1, SDN2, and SDN3. SDN1 method relies upon error-prone repair of DSBs introduced by SDNs through NHEJ, resulting in the deletion or addition of nucleotides. SDN2 and SDN3, in contrast to SDN1, rely on the repair of DSBs through HR. In the case of SDN2, a donor DNA (externally supplied DNA template) carrying a sequence of interest is used to facilitate HR resulting in gene modification at a predetermined site. In the case of SDN3, the donor DNA often contains an insert as big as a transgene (Podevin et al., 2013). The main limitation with SDN2 and SDN3 is low HR frequencies (Pattanayak et al., 2011; Van Vu et al., 2019). Overexpressing proteins facilitating HR can increase the HR frequency. Expressing prokaryotic proteins: RecA (a homolog of eukaryotic RAD51) (Reiss et al., 1996) and RuvC [structure-specific endonuclease (SSE)] increased the HR frequency in plants (Shalev et al., 1999). Interestingly, plants have many endogenous SSEs (GEN1, SEND1, MUS81-EME1, and SLX1), the over-expression of which could be used along with SDNs to increase the efficacy of exchange of gene segments with the donor sequence. An increased HR activity will also facilitate the repair of off-target cleavages (another limitation of SDNs) through HR instead of NHEJ; therefore, reducing the toxicity associated with SDNs. Even though the use of SSEs with SDNs appears to be an attractive approach to direct gene modification/gene incorporation at a specific site, there are limitations of using SSEs. Studies from prokaryotes, yeast, and humans indicate that SSEs can be harmful if not regulated because of their potential to cleave genomic DNA indiscriminately, resulting in genotoxicity (Minocherhomji and Hickson, 2014; Dehe and Gaillard, 2017). SSEs from plants are poorly characterized in terms of their regulation of the activity and substrate specificities, therefore, pausing a major hurdle in using them at the present moment to assist SDNs. Structural and biochemical characterization of plant-specific SSEs is indispensable in fully understanding the molecular basis of HJ resolution, substrate specificities, mapping of cleavage patterns, and regulation of the catalytic activity before they could be implemented in any crop improvement technique.

### Computational Systems Biology and Precision Agriculture

The past decade has witnessed a surge of data science techniques involving a strong component of digital inferences for precision agriculture, that take into account the whole system instead of individual genes or proteins. One of the essential tasks of systems biology is to create explanatory and predictive models of complex systems encompassing important physiological processes. The DRRs offer an untapped opportunity to explore the extent to which known genes and complexes can be used to predict the occurrence, distribution, regulation, and evolution of this machinery across wild and cultivated crop varieties. About 92 fully sequenced and annotated plant genomes are currently available in the Phytozome (Goodstein et al., 2012), in addition to over one thousand vegetative transcriptomes in the public domain (One Thousand Plant Transcriptomes, 2019), as well as an exponentially increasing time-resolved and condition-specific gene expression datasets (both microarrays and RNA-Seq). The next-generation sequencing (NGS) and other high throughput (HTP) experimental datasets have paved the way for reconstructing direct or indirect regulatory connections between various genes and gene products. Transcriptional regulatory inferences from genomic datasets of the known DRR genes across the plant kingdom will enable identification of novel genes and regulators in DRR pathways and the reconstruction of gene regulatory networks that can provide insights into the biological process of DNA repair and recognition.

## DNA Repair and Recombination Proteins as Potential Tools in Crop Improvement

An understanding of various proteins participating in different pathways of DRR has potential to contribute significantly to crop improvement by targeting endogenous plant proteins. In some cases, this information has already translated to model crop plants. This section provides an update regarding the available information on selected proteins participating in DRR and that have the potential for crop improvement (Table 3).

### Photolyase

Photolyase catalyzes the light-dependent direct reversal of the UV induced lesions through photoreactivation. Photolyases are flavoproteins (FAD cofactor) belonging to the photolyase (PHR)/Cryptochrome (CRY) family (Ozturk, 2017). Members of this family are present in bacteria to humans and perform diverse functions. PHR proteins participate in DNA repair, whereas CRY proteins regulate plant development (Liu et al., 2011), and associated with biological rhythms in both plants and animals (Thresher et al., 1998; Vitaterna et al., 1999; Cashmore, 2003). Photolyase based DNA repair is, however, absent in placental mammals (Essen and Klar, 2006). Photolyases belong to two classes: CPD photolyases (substrate: CPDs) and (6-4) photolyases (substrate: (6-4) photoproducts). Structurally, CPD photolyases belong to two subclasses: Class I and Class II CPD photolyases. Plants have two types of photolyases: Class II CPD photolyase and (6-4) photolyase. Interestingly, (6-4) photolyase are closer to Class I CPD photolyase irrespective of different substrate specificities. On the other hand, Class I and Class II CPD photolyases provide the case of convergent evolution (Hitomi et al., 2012).

**FIGURE 2:**
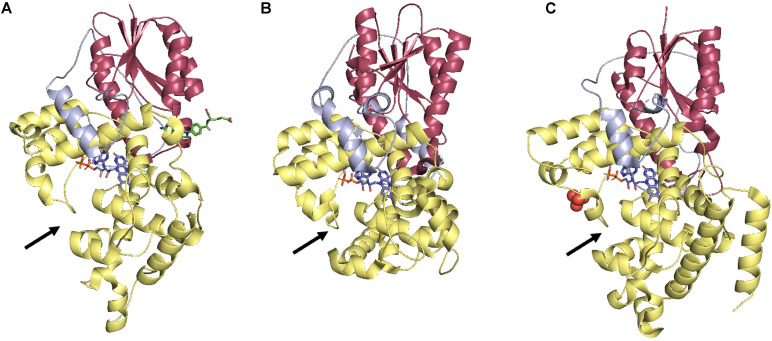
Comparison of photolyases from three different classes. **(A)** Class I CPD photolyase from *E. coli* (PDB: 1DNP), **(B)** Class II CPD photolyase from *O. sativa* (Sasanishiki) (PDB: 3UMV), and **(C)** (6-4) photolyase from *A. thaliana* (PDB: 3FY4). N-terminal α/β domain is shown in Red, C-terminal α-helical domain is shown in Yellow, and interdomain loop is shown in Blue color. The arrows demarcate the substrate binding pocket. FAD molecules are shown in lines in a U-shaped conformation. All the figures were generated using the PyMOL Molecular Graphics System, Version 2.0 Schrödinger, LLC.

Photolyases are by far the most well-studied and structurally characterized DRR proteins in plants. The structures of proteins from all three classes from diverse organisms are available. In general, the overall structure of photolyases shows conservation across all three classes. All photolyases have an N-terminal α/β domain and a C-terminal FAD-binding helical domain. A long linker connects both domains. FAD binds in an unusual U-shaped conformation bringing isoalloxazine and adenine rings in proximity (Park et al., 1995; Hitomi et al., 2009, 2012; Figure 2). A Trp electron transfer pathway restores PHR activity by reduction of FAD in all the three classes. Substrate binding requires flipping of the photoproduct into the photoproduct binding cavity. Amino acid residues from PHR further stabilize the complementary undamaged strand. Although Class I CPD photolyases and (6-4) photolyases are evolutionarily related, there are significant differences in the size of photoproduct binding site (active site of (6-4) photolyase being narrower), mode of catalysis [(6-4) photolyase requires two His for catalysis], and second cofactor binding site (Hitomi et al., 2009). Class II CPD photolyase has low sequence similarity with class I CPD photolyase, but it still adopts the same overall fold. Marked differences are present in the C-terminal substrate-binding region and the mode in which proteins from the two classes interact with the DNA (Hitomi et al., 2012). Class II CPD photolyase structure provides clues regarding how a single amino acid substitution can improve the UV resistance of certain strains of rice varieties over others. The more UV tolerant rice cultivars have Gln296 in class II CPD photolyase, while less tolerant cultivars have His at this position. Gln indeed helps stabilize the overall structure of the protein (Hitomi et al., 2012). Norin 1 is one of the most widely cultivated varieties of rice is UV sensitive because of single Gln to Arg change at position 126, resulting in a less stable photolyase (Hidema et al., 2000). Both these examples provide classic examples of structure-based understanding to engineer the photolyases for crop improvement.

### DNA Glycosylases

**FIGURE 3:**
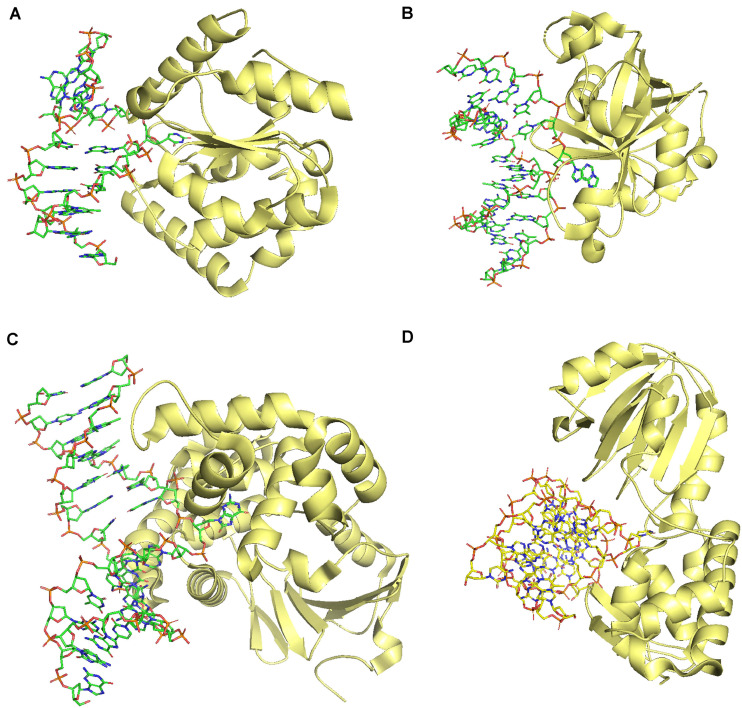
Comparison of DNA glycosylases from four different super families showing a common mechanism of catalysis by base flipping. **(A)** UDG superfamily (PDB: 1EMH), **(B)** AAG superfamily (PDB: 1F4R), **(C)** HhH superfamily (PDB: 2NOI), **(D)** H2TH superfamily (PDB: 1R2Z). All the figures were generated using the PyMOL Molecular Graphics System, Version 2.0 Schrödinger, LLC.

DNA glycosylases catalyze the first reaction in BER. There are two types of DNA glycosylases: monofunctional and bifunctional DNA glycosylases. Monofunctional glycosylases cleave only N-glycosidic bonds resulting in an AP site. In contrast, bifunctional glycosylases cleave the N-glycosidic bond and phosphodiester bond (Roldan-Arjona et al., 2019). Bifunctional DNA glycosylases possess an AP lyase activity in addition to the DNA glycosylase activity. Monofunctional DNA glycosylases, in contrast, are devoid of AP lyase activity. Therefore, an AP endonuclease activity follows catalysis by monofunctional DNA glycosylases (Fromme et al., 2004). DNA glycosylases exhibit a wide range of substrate specificities. Almost all eukaryotic DNA glycosylases rely on flipping the damaged or modified base into the active site, followed by the cleavage of the N-glycosidic bond (Figure 3). There are five structural superfamilies of plant DNA glycosylases: Uracil DNA glycosylases (UDG), Alkyladenine DNA glycosylase (AAG), helix-hairpin-helix (HhH), and helix-two-turn-helix (H2TH) (reviewed in Roldan-Arjona et al., 2019).

#### Uracil DNA Glycosylases (UDG)

Uracil DNA glycosylases (UDG) remove Uracil from DNA. UDG superfamily encompasses six subfamilies (Brooks et al., 2013; Roldan-Arjona et al., 2019). Only Family-1 of UDG is present in plants (Talpaert-Borle and Liuzzi, 1982; Warner, 1983; Bensen and Warner, 1987; Bones, 1993). *At*UNG is a UDG isolated and characterized from *A. thaliana* (Cordoba-Canero et al., 2010). Family-1 of UDG, as exemplified by human UDG, comprises a central four-stranded parallel β-sheet, flanked by eight α-helices (Figure 3A). These are monofunctional enzymes. Breaking of N-glycosidic bond involves flipping out of the base, followed by the distortion and weakening of the N-glycosidic bond before the cleavage (Parikh et al., 2000). At present, no structural information is available on plant UDG. Plant UDG active site is more specific and selective compared to UDG from other organisms because of the inability of *At*UNG to act upon 5-fluorouracil (5-FU). *Atung* mutant plants do not show any apparent defect; however, inactivation of *At*UNG protects the plant against the cytotoxic effects of 5-FU (Cordoba-Canero et al., 2010).

#### Alkyladenine DNA Glycosylases

Alkyladenine DNA glycosylases (AAG) or alkylpurine glycosylases are monofunctional enzymes and remove alkylated purines. Alkylated purines may arise because of cellular metabolic processes, mutagenic agents, or environmental mutagens. Alkylated purines may be cytotoxic (3-methyladenine), mutagenic (O^6^-methylguanine), or harmless (7-methlylguanine) (Loechler et al., 1984; Larson et al., 1985; Santerre and Britt, 1994). AAG has been extensively characterized biochemically and structurally from humans (hAAG), bacteria, and archaea (Brooks et al., 2013). hAAG cleaves a variety of alkylated bases (adenine and guanine). hAAG is a single domain protein with a core of eight β-strands forming a curved antiparallel β-sheet and a β-hairpin that protrudes into the minor groove of DNA. Flanking loops and α-helices form the remaining DNA binding surface (Lau et al., 1998). The β-hairpin intercalates into the minor groove and flips the modified nucleotide into the active site. A water molecule then makes a nucleophilic attack to cleave the N-glycosidic bond (Figure 3B). A combination of the shape, hydrogen-bonding capability, and aromaticity of the modified base dictate the selectivity of AAG towards the damaged nucleotide (Lau et al., 1998, 2000). Incidentally, alkylating agents are the most used mutagens to induce mutations for crop improvement, and still, our understanding regarding the plant AAG is in infancy. 3-methyladenine glycosylase is an AAG from *A. thaliana* (Santerre and Britt, 1994) and requires further structural and biochemical characterization.

#### HhH Superfamily

Helix-hairpin-helix superfamily comprises of both monofunctional and bifunctional enzymes. They repair a wide range of modified bases resulting from alkylation, oxidation, or spontaneous deamination. The structure consists of N-terminal and C-terminal α-helical domains, connected by a type II β-hairpin (Figure 3C). Various homologs of *E. coli* DNA glycosylases acting upon alkylated purines and oxidized bases are present in Arabidopsis (Bjelland et al., 1993; Labahn et al., 1996; Britt, 2002; Roldan-Arjona et al., 2019). Members of this family involved in excising oxidized bases have also been identified and characterized from Arabidopsis. A bifunctional OGG1 protein (8-oxyguanine DNA glycosylase) from Arabidopsis and *Medicago truncatula* acts upon 7-hydro-8-oxoguanine (8-oxoG) (Roldan-Arjona et al., 2000; Dany and Tissier, 2001; Macovei et al., 2011). A monofunctional MBD4-like glycosylase (*At*MBD4L) from Arabidopsis excises uracil or thymine mispaired to guanine (Ramiro-Merina et al., 2013). Further characterization on these glycosylases is required to understand the substrate specificities, and physiological phenotypes arising from the defects in these proteins.

Plants exclusively contain 5-meC DNA glycosylases. They form a separate family of glycosylases (**DEMETER-LIKE (DML) family)** of HhH superfamily (Zhu, 2009; Brooks et al., 2013; Roldan-Arjona et al., 2019). DML family comprises of proteins like DME (DEMETER) (Choi et al., 2002), Ros1 (Repressor of silencing 1) (Gong et al., 2002), DML2 (DME-like 2), and DML3 (DME-like 3) (Ortega-Galisteo et al., 2008) from *Arabidopsis*. All the members of this family are bifunctional enzymes. They take part in crucial processes like regulation of transcription and inhibition of erroneous gene silencing by demethylating DNA through the process of BER (Choi et al., 2002; Gong et al., 2002; Ortega-Galisteo et al., 2008). At present, no structural information regarding the members of the DML family is available except for sequence analysis and modeling studies (Ponferrada-Marin et al., 2011). The salient features distinguishing members of DML family from other members of HhH superfamily are the presence of a [4Fe-S] cluster, a discontinuous catalytic site, and an additional Lys rich N-terminal domain and a C-terminal domain (Ponferrada-Marin et al., 2011). Structure-based studies of these proteins are essential for understanding substrate specificities and catalytic mechanisms of these proteins.

#### H2TH Superfamily

Helix-two-turn-helix superfamily members are primarily involved in repairing oxidative damages. *E. coli* Formamidopyrimidine DNA glycosylase (Fpg/MutM) and Endonuclease VIII (Nei) are the typical members of H2TH superfamily (Roldan-Arjona et al., 2019). Members of this superfamily are bifunctional enzymes. They remove a wide range of modified bases, *e.g.*, 5-hydroxyuracil, 5-hydroxycytosine, dihydrouracil, thymine glycol, *etc.* (Sugahara et al., 2000; Kathe et al., 2009; Zhu et al., 2016). Structurally, members of this family comprise an N-terminal domain and a C-terminal domain containing a Zn finger. A flexible hinge connects the two domains (Sugahara et al., 2000; Figure 3D). The catalysis involves tautomerization-dependent recognition, flipping of the base, followed by excision (Zhu et al., 2016). Plants have homologs of *E. coli* Fpg with Arabidopsis having seven different isoforms (Ohtsubo et al., 1998). More structural and biochemical characterization of plant FPG proteins are essential to ascertain their role in repairing oxidative damage. More studies are required to determine the relative contributions of plant FPG proteins and OGG1 in oxidative damage repair (Cordoba-Canero et al., 2014).

Since most of the commonly used mutagens, modify the nitrogen bases, which are subsequently repaired by DNA glycosylases, a structure-based study of these DNA glycosylases becomes of utmost importance for increasing the efficacy of mutagenesis during crop improvement. Structural and biochemical studies will provide mechanistic insights into the repair of bases modified by chemical mutagens, amino acid residues contributing to substrate specificities, and overall stability of the proteins. Such studies will enable engineering DNA glycosylases with variable substrate specificities and catalytic mechanisms, providing more control over mutagenesis. Structure-based rational engineering had shown changing substrate specificity and transforming a DNA glycosylases into a site-specific DNA glycosylase (Kwon et al., 2003). DNA glycosylases also play a role in epigenesis through demethylation with a strict preference for 5-methylcytosine (5-meC) over thymine in the CpG sequence context (Morales-Ruiz et al., 2006; Table 3). Structure-based engineered DNA glycosylases can alter demethylation and therefore has potential in epigenetic studies.

### Structure-Specific Endonucleases (SSEs)

Endonucleases have played a significant role in crop improvement. Engineered artificial nucleases (ZFN, TALEN, and CRISPR-Cas) are in use for the last two decades (Zhang et al., 2017, 2018; Xu et al., 2019). All these nucleases introduce DSBs in the target gene, which is then repaired by plant internal DRR machinery. Structure-specific endonucleases (SSEs) are among the key players involved in DSB repair. Unlike sequence-specific endonucleases, SSEs recognize the secondary structure of DNA. During DNA metabolism, repair, and recombination, various joint DNA molecules (e.g., 5′ flap, 3′ flap, replication forks, splayed arm DNA, Holliday junctions, *etc.*) appear as intermediates. SSEs process these joint DNA molecules and restore regular DNA duplexes. While engineered nucleases are in use extensively to generate DNA breaks in a sequence-specific manner, knowledge about the structure of SSEs will enhance understanding regarding DRR in plants. Besides, it will provide an additional toolkit for designing innovative methods for crop improvement in the future. Various SSEs participate in different DRR pathways in plants: FEN1, GEN1, SEND1, MUS81-EME1, and SLX1.

#### Flap Endonuclease 1 (FEN1)

**FIGURE 4:**
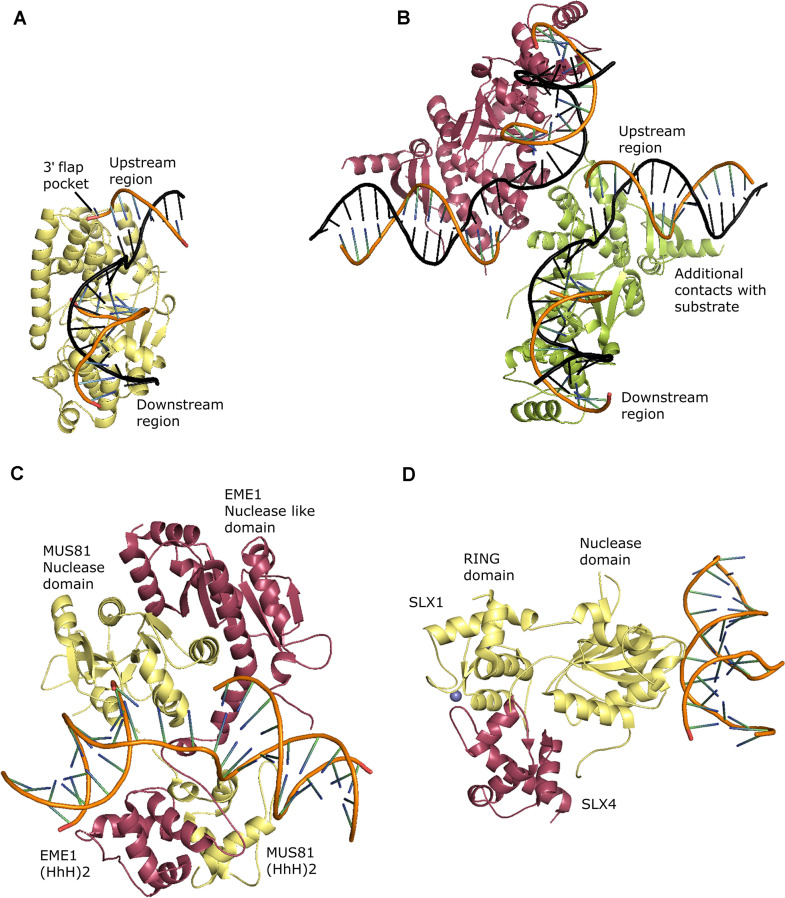
Structure specific endonucleases. **(A)** Crystal structure of human FEN1 in complex with a 5′ flap substrate: non-nicked DNA strand is shown in black color (PDB: 3Q8M), **(B)** Crystal structure of *Chaetomium thermophilum* GEN1 in complex with product DNA: two symmetry related monomers are shown in red and green colors and the non-nicked DNA strand is shown in black color (PDB: 5CO8), **(C)** Crystal structure of human MUS81-EME1 in complex with a 3′ flap substrate: MUS81 and EME1 are shown in yellow and red colors respectively (PDB: 4P0R), **(D)** Crystal structure of *Thermothielavioides terrestris* SLX1-SLX4^*CCD*^ in complex with a distorted DNA duplex presenting one of the three DNA binding sites of SLX1: SLX1 and SLX4^*CCD*^ are shown in yellow and red colors respectively, the Zn ions of RING domain are shown in blue color (PDB: 6SEI). All the figures were generated using the PyMOL Molecular Graphics System, Version 2.0 Schrödinger, LLC.

Flap Endonuclease 1 (FEN1) belongs to the Rad2/XPG family of nucleases. Members of the RAD2 family are involved in DNA replication (FEN1), repair (FEN1, XPG, EXO1), and recombination (GEN1, SEND1)(Lieber, 1997; Furukawa and Shimada, 2009; Tsutakawa et al., 2011). FEN1 has endonuclease as well as 5′-3′ exonuclease activities. FEN1 takes part in the removal of 5′ flap intermediates during long patch BER (Huggins et al., 2002; Asagoshi et al., 2010; Sun et al., 2017) and processing of Okazaki fragments during replication by getting associated with PCNA (Gomes and Burgers, 2000; Liu et al., 2004; Dovrat et al., 2014). FEN1 comprises a catalytic domain, and a flexible PCNA interacting C-terminal domain. The crystal structure of human FEN1 in complex with PCNA provides clues regarding the sliding of FEN1 along with PCNA in an inactive conformation and switching to an active conformation on encountering a single-stranded flap DNA (Sakurai et al., 2005). The crystal structure of the catalytic domain of human FEN1 in complex with branched substrates and structural comparison with unliganded FEN1 provides insights into the structural attributes that allow FEN1 to process branched DNA structures (Kim et al., 2001; Sakurai et al., 2005; Tsutakawa et al., 2011). FEN1 has two separate DNA duplex binding sites on its surface (downstream region and upstream region) (characteristic of Rad2/XPG family), which allows DNA bending and therefore facilitate interaction with either branched or nicked DNA. An H2TH motif interacts specifically with the non-nicked strand (or template strand), thus positioning the DNA substrate in a catalytically favorable orientation. Structural features that confer specificity towards flap substrates are the presence of a 3′ flap pocket and a gateway that allows only a single-stranded DNA to pass through it and enters the catalytic site. Catalysis involves double-base flipping and a 2-metal ion active site (Tsutakawa et al., 2011; Figure 4A). The characterization of FEN1 from plants is inadequate. FEN1 from *Brassica oleracea*, *Arabidopsis thaliana*, *Oryza sativa*, and *Triticum vulgare* participate in DNA repair, cell growth, organ formation, maintenance of genome stability, and transcriptional gene silencing (Gallego et al., 2000; Kimura et al., 2000; Przykorska et al., 2004; Zhang J. et al., 2016). Similar to other eukaryotes, PCNA appears to coordinate DNA repair and replication by interacting with FEN1 (Kimura et al., 2001). *Arabidopsis* SAV6 is a FEN1 homolog with an endonuclease activity but lacking an exonuclease activity (Zhang J. et al., 2016). In plants, so far, structural, and biochemical studies have not been done on FEN1. However, crystals of AtPCNA in association with PIP (PCNA interacting protein box motif) from FEN1 have recently been obtained (Kowalska et al., 2020). Structural studies on plant FEN1 will further shed light on the role of FEN1 in coupling DNA repair and replication in plants.

#### GEN1

GEN1 is an HJ resolvase that works in a pathway parallel to SLX1 and MUS81-EME1 mediated HJ resolution (Wyatt et al., 2013). GEN1 belongs to the Rad2/XPG nuclease family and, therefore, structurally related to FEN1 (Ip et al., 2008). GEN1 can process HJs by introducing two symmetrical nicks across the junction and 5′ flaps by cleaving the single-stranded flap. GEN1 can also process replication forks. Unlike FEN1, GEN1 does not have an exonuclease activity (Rass et al., 2010). Like FEN1, GEN1 is monomeric in solution; however, it dimerizes on binding an HJ to facilitate two symmetrical nicks during HJ resolution (Rass et al., 2010). GEN1 structures reveal features common to the members of the Rad2/XPG family: two metal ion catalysis mechanism, two DNA binding surfaces separated by a wedge allowing bending of DNA, and an H2TH motif involved in binding the non-cleaved strand. Nevertheless, GEN1 has specialized structural features that enable GEN1 to dimerize and process HJs. The helical arches forming the gateway in FEN1 are modified to recognize the central portion of an HJ and provide a surface for GEN1 dimerization (Figure 4B). Relaxation of HJ and transition of GEN1 active site from disordered to ordered state appear to regulate the activity of GEN1 (Lee et al., 2015; Liu et al., 2015). A chromodomain, a structural feature exclusive to GEN1, further stabilizes the interaction between GEN1 and HJ. Truncation in chromodomain affects nuclease activity (Lee et al., 2015). Plants have two homologs of GEN1: GEN1 and SEND1 (Single-Strand DNA Endonuclease1) (Furukawa et al., 2003; Moritoh et al., 2005; Bauknecht and Kobbe, 2014). AtGEN1 and AtSEND1 from *A. thaliana* can resolve HJs by two symmetrical nicks; however, both the proteins have distinct substrate specificities guided by the structure and sequence of the substrate (Bauknecht and Kobbe, 2014). OsGEN1 can also process an HJ by two symmetrical nicks without any cooperativity in the two nicking events (unlike other known FEN1) (Yang et al., 2012). AtSEND1 (and not AtGEN1), along with MUS81 from *A. thaliana* is essential for telomere stability (Olivier et al., 2016). Interestingly, in rice, OsGEN1 (and not OsSEND1) plays an essential role in homologous recombination (Wang et al., 2017). More structural insights are imperative to understand HJ resolution by GEN1 and SEND1 from plants and to pinpoint the structural elements responsible for differences in the functions of these two proteins.

#### MUS81

MUS81 forms a heterodimeric complex with a non-enzymatic partner, EME1 (Figure 4C). Both MUS81 and EME1 belong to the XPF family of SSEs (Ciccia et al., 2008). MUS81-EME1 complex plays an important role in resolving HJs, rescuing collapsed replication forks, DSB repair, and interstrand crosslink repair (Boddy et al., 2001; Doe et al., 2002; Hanada et al., 2006). Biochemically, MUS81-EME1 can process nicked HJ (nHJ), 3′ flaps, and replication forks (Fricke et al., 2005). nHJs are the preferred substrates for MUS81-EME1 in comparison to intact HJ (iHJ). MUS81-EME1 coordinates with SLX1-SLX4 complex to resolve HJs by a nick and counter nick mechanism, where SLX1-SLX4 makes the first nick in the iHJ generating an nHJ, which serves as a substrate for MUS81-EME1 (Gaillard et al., 2003; Svendsen et al., 2009; Wyatt et al., 2013). Crystal structure of truncated MUS81-EME1 (N-terminal region removed from both the proteins) complexes from human in unliganded and in complex with DNA substrates provided comprehensive insights into the overall architecture and substrate preferences of MUS81-EME1 (Chang et al., 2008; Gwon et al., 2014). Both MUS81 and EME1 comprises of a nuclease domain and two repeats of the helix-hairpin-helix motif (HhH)_2_ connected by a linker. The nuclease domain of EME1 (also referred to as nuclease-like domain) is catalytically dead. The domains from both the proteins intertwine with each other. The differences in the linkers result in a quaternary structure, distinct from the other members of the XPF family (Kim et al., 2008). (HhH)_2_ domains of both MUS81 and EME1 along with the nuclease domain, play a significant role in binding branched substrates. Substrate binding induces conformational changes in the overall structure of MUS81-EME1, resulting in exposing a hydrophobic wedge and a 5′ end binding pocket. These structural changes are essential for bending the DNA substrate, substrate specificity (nHJ or a 3′ flap), and catalysis (Figure 4C; Gwon et al., 2014). In plants, homologs of MUS81, as well as EME1, have been identified from *Arabidopsis* and rice (Berchowitz et al., 2007; Higgins et al., 2008; Mimida et al., 2007; Geuting et al., 2009). Arabidopsis genome has two homologs of EME1: AtEME1a and AtEME1b. Both EME1 homologs are capable of forming a functional enzymatic complex with MUS81 with differences in the processing of iHJ (Geuting et al., 2009). Interestingly, the *OsMUS81* gene of rice produces two alternate transcripts: *OsMUS81α* and *OsMUS81β* differing in the HhH motif at the C-terminal end. Further studies are necessary to characterize MUS81-EME1 complexes from plants.

#### SLX1

SLX1 is a member of the GIY-YIG family of endonucleases (Dunin-Horkawicz et al., 2006). In fungi and animals, SLX1 forms a heterodimeric complex with a non-enzymatic protein, SLX4. SLX1 interacts with SLX4 through the CCD domain (C-terminal conserved domain) of SLX4 (Fekairi et al., 2009; Gaur et al., 2015). SLX1-SLX4 complex participates in HR and maintenance of telomeres. Biochemically, SLX1-SLX4 complex can cleave HJs, 5′ flap, replication forks, 3′ flaps, and splayed arm DNA substrates (Fricke and Brill, 2003; Gaur et al., 2015, 2019). Crystal structures of SLX1 and SLX1 in complex with CCD domain of SLX4 (unliganded and in complex with DNA substrate) provided insights into the regulation of SLX1 activity and substrate specificity. SLX1 comprises an N-terminal GIY-YIG nuclease domain, and a C-terminal RING domain connected by a long α-helix (Gaur et al., 2015, 2019). In fungi, SLX1 exists in a self-inhibitory homodimeric form. An interaction between SLX1 and SLX4 is critical for the enzymatic activity of SLX1 (Gaur et al., 2015). Unlike other SSEs, SLX1 is devoid of any DNA binding secondary structural features. Instead, SLX1 has DNA binding patches on its surface. SLX1 uses the spatial organization of these DNA binding patches to bend the DNA substrate and identify the branching point as a flexible discontinuity in branched DNA substrates (Figure 4D; Gaur et al., 2019). Based on the available sequences, a GIY-YIG containing, SLX1 like protein called HIGLE has been identified in Arabidopsis (Cho et al., 2017). SLX4 has not been identified in plants so far and is an overly exciting avenue for future research. Further studies are required to ascertain the role of plant SLX1 in HJ resolution.

### RTR Complex (RECQ4A-TOP3α-RMI1)

Homologous recombination is a central event during a crop breeding event. The key intermediate of HR is HJ (a four-way DNA junction). There are two independent mechanisms of processing an HJ: resolution and dissolution. Dissolution of HJ involves a complex of RecQ helicase (RECQ4A), topoisomerase 3α (TOP3α), and a structural protein RMI1 (Bagherieh-Najjar et al., 2005; Hartung et al., 2007a; Knoll and Puchta, 2011). Similar complexes from humans (BTR complex: BLM helicase, Topoisomerase 3α, RMI1, and RMI2), and yeast (Sgs1, Top3, and Rmi1) provide insights into the molecular architecture and function of RTR complex from plants (Wu L. et al., 2005; Bachrati and Hickson, 2009; Cejka et al., 2010; Bizard and Hickson, 2014). In humans, mutations in BLM helicase result in Bloom syndrome, characterized by increased frequency of sister chromatid exchange (Ellis et al., 1995). A similar phenotype is associated with mutations in yeast Ssg1 (Miyajima et al., 2000). Among plants, mutations associated with various components of the RTR complex result in hypersensitivity to DNA damaging agents and accumulation of unrepaired DSBs (Knoll et al., 2014). RTR complex by participating in dissolution significantly decreases the probability of reshuffling DNA segments by crossing over. RTR complex is one of the critical negative regulators of crossing over along with MMR proteins (Choi, 2017). A disruption of the MSH2 gene has already shown a 40% increase in the crossover rate in *Arabidopsis* (Emmanuel et al., 2006). Similar studies involving structural and biochemical characterizations for plant RTR complex are necessary to open new avenues for crop improvement.

### TLS Polymerases

Trans-lesion DNA synthesis polymerases are essential for plant survival as they restart the stalled replication fork. TLS is an error-prone mechanism of tolerating DNA lesions. TLS can have both mutagenic and less mutagenic activities in plants (Sakamoto, 2019). Disruptions of AtPol ζ or AtRev1 decrease homologous recombination frequencies, whereas disruption of AtPol η increases the frequencies of homologous recombination in somatic tissues (Sakamoto, 2019). An overexpression of AtPOLH (coding for AtPolη) increases UV resistance in *Arabidopsis* (Jesus Santiago et al., 2008). REV3 subunit of Pol ζ appears to cooperate with structure-specific endonuclease, MUS81-EME1 (a participant in HJ resolution), however, similar cooperation with RECQ4A (a participant in HJ dissolution) is not known (Kobbe et al., 2015). All these studies have immense potential in designing strategies for crop improvement. The field of plant TLS polymerases is in initial phases. There is much to be done to understand substrate specificities of various plant TLS polymerases, regulation of their expression, coupling with DNA synthesis, and crosstalk with other DRR mechanisms such as photoreactivation, BER, NER, and HR.

#### Final Considerations

An intricate interplay of various DRR mechanisms continuously repairs DNA damages arising from multiple internal and external DNA damaging agents. Complex crosstalk between different DRR pathways exists in plants. The different DRR pathways are under regulation in the cell-cycle, tissue-specific, and development stage-dependent manner. Incidentally, factors described above likewise impact the success of present-day crop-improvement techniques. Therefore, an understanding of these pathways could further assist in fine-tuning various gene-editing techniques. There are already some attempts to understand the complex crosstalk between various DRR pathways, especially in the context of HR. *e.g.*, the nature of DSB ends, and the phase of the cell cycle governs the choice between the two pathways involved in DSB repair: HR and NHEJ (Symington and Gautier, 2011). Plant hormones further control the choice between the two pathways. Abscisic acid increases the frequency of HR, while at the same time, suppresses Ku70 (a vital component of NHEJ) (Yin et al., 2009). Interestingly the two pathways are not entirely independent of each other, and various gene rearrangements could be explained based on the cooperative actions of both the pathways (Gorbunova and Levy, 1999). Various environmental factors (*e.g.*, the chemical composition of soil) and different amounts of exposure to mutagen can also influence the frequency of HR (Kovalchuk et al., 2000). Besides crosstalk with NHEJ, HR also crosstalks with the MMR pathway through MSH4 and Rad51 (Higgins et al., 2004). An interconnection between HR and early steps of the NER pathway also exists and mediated by CENTRIN2 (Molinier et al., 2004). A similar level of complexity involves the repair of DNA lesions induced by UV radiation and chemical mutagens. Various pathways ranging from photoreactivation, BER, MMR, NER to the involvement of TLS DNA polymerases cooperate/compete to repair a wide array of DNA lesions. There are indications of crosstalk between BER and other DDR pathways involving post-translational modifications (Limpose et al., 2017). OGG1, a glycosylase that repairs oxidized bases through BER, interacts with HR protein RAD52 with functional implications. This interaction inhibits RAD52 while increasing the turnover rate for OGG1 (de Souza-Pinto et al., 2009). All these studies imply the complex nature of plant DRR, and a necessity to comprehend these complexities for transforming future crop improvement techniques.

Crop improvement methodologies rely on generating DNA lesions through irradiation or chemical mutagenesis, the integration of foreign nucleic acids, and the repair of DSBs generated by site-directed nucleases. The success of any technique involved in crop improvement relies on the intrinsic DRR machinery of the plants and a complex interplay between various DRR pathways. There is an increased emphasis on increasing the crossover frequencies by manipulating the existing crosstalk between different DRR pathways (Blary and Jenczewski, 2019). One of the most successful methods of introducing mutations in a plant is the use of chemical mutagens. Such mutations by-and-large impact single nucleotides that are repaired by various glycosylases. Future crop improvement methodologies could be designed using engineered DNA glycosylases based on in-depth structural, biochemical, and computational studies. A structural-based study of different endonucleases from sources other than plants (*e.g.*, robust tools like CRISPR/Cas) enabled researchers to induce site-specific DSB successfully. Plants have several endogenous structure-specific endonucleases already involved in DRR pathways. The structural mechanism of the catalysis and regulation of endogenous structure-specific endonuclease can unleash their potential for crop improvement. Studying various aspects of DRR, therefore, provides an excellent opportunity to improvise the existing methods or to innovate new ways for efficient and faster crop improvement to meet the demand of billions of humans in an environmentally friendly manner.

## Author Contributions

PV, RT, GY, and VG contributed significantly to various sections of the manuscript. VG conceived the idea of the manuscript. All authors contributed to the article and approved the submitted version.

## Conflict of Interest

The authors declare that the research was conducted in the absence of any commercial or financial relationships that could be construed as a potential conflict of interest.
